# A review of user needs to drive the development of lower limb prostheses

**DOI:** 10.1186/s12984-022-01097-1

**Published:** 2022-11-05

**Authors:** Sabina Manz, Romain Valette, Federica Damonte, Lucas Avanci Gaudio, Jose Gonzalez-Vargas, Massimo Sartori, Strahinja Dosen, Johan Rietman

**Affiliations:** 1 Ottobock SE & Co. KGaA, Duderstadt, Germany; 2 Department of Health Science and Technology, Aalborg University, Aalborg, Denmark; 3 Department of Biomechanical Engineering, University of Twente, Enschede, The Netherlands; 4 Roessingh Research and Development, Enschede, The Netherlands

**Keywords:** User-centered design, Lower limb prosthetics, User needs, Prosthesis requirements

## Abstract

**Background:**

The development of bionic legs has seen substantial improvements in the past years but people with lower-limb amputation still suffer from impairments in mobility (e.g., altered balance and gait control) due to significant limitations of the contemporary prostheses. Approaching the problem from a human-centered perspective by focusing on user-specific needs can allow identifying critical improvements that can increase the quality of life. While there are several reviews of user needs regarding upper limb prostheses, a comprehensive summary of such needs for those affected by lower limb loss does not exist.

**Methods:**

We have conducted a systematic review of the literature to extract important needs of the users of lower-limb prostheses. The review included 56 articles in which a need (desire, wish) was reported explicitly by the recruited people with lower limb amputation (N = 8149).

**Results:**

An exhaustive list of user needs was collected and subdivided into functional, psychological, cognitive, ergonomics, and other domain. Where appropriate, we have also briefly discussed the developments in prosthetic devices that are related to or could have an impact on those needs. In summary, the users would like to lead an independent life and reintegrate into society by coming back to work and participating in social and leisure activities. Efficient, versatile, and stable gait, but also support to other activities (e.g., sit to stand), contribute to safety and confidence, while appearance and comfort are important for the body image. However, the relation between specific needs, objective measures of performance, and overall satisfaction and quality of life is still an open question.

**Conclusions:**

Identifying user needs is a critical step for the development of new generation lower limb prostheses that aim to improve the quality of life of their users. However, this is not a simple task, as the needs interact with each other and depend on multiple factors (e.g., mobility level, age, gender), while evolving in time with the use of the device. Hence, novel assessment methods are required that can evaluate the impact of the system from a holistic perspective, capturing objective outcomes but also overall user experience and satisfaction in the relevant environment (daily life).

## Background

### Effects of lower limb loss

Lower limb loss is a devastating experience with substantial functional and psychological impacts on the everyday life of the affected persons [1–3]. While autonomous mobility and participation in social and professional activities are critical aims in the lives of people with amputation, these goals are challenging to achieve due to limitations in the available functions of current prosthetic technology. Indeed, people with a lower-limb amputation using a prosthetic device may suffer from altered balance and gait control [4–11], which contributes to increased metabolic energy consumption [12–14], as well as long-term comorbidities such as low-back pain [15], osteoarthritis and osteoporosis [16].

The improvement in the quality of life of those affected by limb loss to a level comparable to that of able-bodied people is the ultimate goal [3, 17–19]. The symptoms of anxiety and depression are often observed [20–24], even if they tend to vary between individuals in terms of temporality, intensity [23, 25–27], and cause of amputation [28]. Anxiety and depression correlate significantly with psychosocial, social, and disability adjustments, as well as with body-image disturbances and other factors such as fear of pain [29–33]. Furthermore, the loss of independence is a recurrent issue found in prosthetic users, which has a substantial psychological impact on self-esteem and frustration [34].

The loss of somatosensory feedback from the lower limb, combined with a diminished ability to produce rapid gait adjustments (e.g., voluntary control of the prosthetic limb during gait distortions or perturbations) and reduced lower-limb muscle strength cause an increase in the risk of falling [35–38]. The fear of falling is more commonly present in prosthetic users compared to the general population [39], and it is associated with an increased risk of falls [40].

After an amputation, social interaction is reported to decrease and leisure time activities change significantly, especially in the younger population. Out of 228 individuals with traumatic amputations at a young age, almost half stated that they visit friends and family less frequently, and around two-thirds go less often to the cinema, theatre, sports events, library, and dances. After amputation, over 40% of respondents reported a substantial change in their leisure time activities, while less than 15% still take an interest in the same activities. The most common activities were limited to more passive tasks like reading, watching television, listening to radio/music, and housekeeping [41]. The reasons for not engaging in other activities range from self-consciousness and stigmatization of their conditions to difficulties in locomotion [42, 43]. Young individuals living with an amputation would avoid going swimming in a public pool, dancing, or sunbathing [42], while older individuals reported that shopping and visiting friends were affected [43]. Furthermore, people with amputation aged 50 and older suffer less from severe changes in habits after amputation [44]. Studies have shown that once a person has a visually detectable disability, other people tend to avoid interactions, and this also applies to prosthesis users [45].

### Factors associated with the use of a prosthetic device

The use of a prosthesis during gait and gait-related activities, as well as donning/doffing of the system requires physical skills such as balance and coordination, but also cognitive capacities to learn new skills and adapt them to different scenarios. This can be especially challenging considering that the entire control loop is affected or missing (i.e., control of the leg and sensory feedback) [46–49]. The use and maintenance of a prosthetic leg involve a number of areas related to cognitive processes such as memory, attention, concentration, visuospatial function, and organizational skills [50]. At the same time, cognitive impairment appears to be more prevalent in people with lower-limb amputation than in the general population [51]. The cognitive effort added by the loss of control and perception, alongside the need of relying on visual cues to monitor the prosthesis, results in additional cognitive burden which can interfere with the execution of the tasks and the ability to multitask [9, 52, 53].

Stump pain constitutes one of the main causes of discontinuation of the use of prosthetic legs [54, 55]. Indeed, skin lesions are seen in 63 to 82% of people with lower-limb amputation [56], and shear stresses and pressure distribution exerted by the liner and the socket on the stump will impact the comfort of the device. Stump pain can be classified as intrinsic and extrinsic [57]. Extrinsic stump pain is closely related to residual limb health, for instance, pressure sores, or allergic and irritant contact dermatitis [58]. A common form of intrinsic stump pain seen in people with lower limb amputation is phantom limb pain [59]. Users experiencing phantom limb or residual limb pain are less satisfied and have difficulty adjusting to their disability compared to those who do not have pain, while the activity restriction remains at a similar level [60].

### Motivation for this review

An effective prosthesis can allow those affected by limb amputation to gain back the lost functionality, personal health, participation in society, and thereby improve the overall quality of life. However, the above-described challenges, and the increasing prevalence of lower limb amputations with increasing age [61], highlight the importance of paying attention to specific needs expressed by the users during the development of new, more advanced devices. User needs are hereby defined as the requirements directly expressed by the prosthetic users themselves and can relate to different areas of their lives (e.g., daily activities, social engagement, etc.). While reviews of user needs for people with upper limb amputation already exist [62], to date, there is no published review summarizing user needs comprehensively, across lower limb amputation levels and different aspects of subjective experience (e.g., functional, psychological, ergonomics etc.). Therefore, this review aims to summarize those multifaceted needs and requirements for them to be translated into the development process of lower limb prosthetic systems. This will provide an extensive collection of user needs that reflect the opinions of prosthetic device users. Such review could potentially facilitate the design of user-tailored prosthetic systems, which will lead to further positive impacts on the lives of those affected by limb loss, including higher satisfaction and use rates.

## Methods

A comprehensive literature search has been performed on the PubMed and MEDLINE databases. The search terms (and their combinations) were: (satisf* OR (quality of life) OR (user) OR (need*) OR (well being)) AND (prosth* OR amput*) AND ((lower limb*) OR leg OR legs) NOT (dental*) NOT (ortho*). Results published in the English language until July 2022 were considered for this review. Additional articles from the reference lists of the search results have been considered as well. The inclusion criteria for the selection of articles from the search results was that they were studies on lower limb prosthetic users expressing specific user needs, identified by subjective feedback from the users (e.g., questionnaires).

**Fig. 1:**
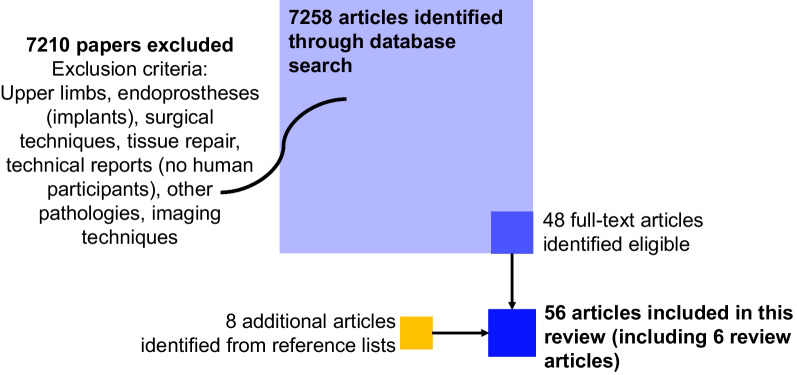
Flowchart of inclusion and exclusion of articles during the systematic search process

The search resulted in 7258 articles (including duplicates). After removing duplicates, and reviewing title and abstract information for relevance according to the above-mentioned inclusion criteria, 7210 articles were excluded as being not relevant. The papers were excluded based on the exclusion criteria in Fig. 1. The remaining 48 articles were read carefully [1, 27, 34, 44, 52, 60, 63–104] and an additional 8 articles were identified as relevant for this review (from the reference lists of the 48 articles) [17, 21, 54, 105–109]. These articles were not found during the initial database search but they included prosthetic device requirements expressed by the users and were therefore included in this review. As a result, 56 articles were considered, including six review articles [27, 75–77, 89, 90]. These review articles either focused on a smaller user group, for instance, transtibial amputations [75] or traumatic amputations [77], or specific types of needs only (i.e., psychosocial or physical aspects) [27, 76, 89, 90]. Consequently, they do not reflect a complete image of the needs of a user with lower limb amputation, but only a subset of needs. Figure 1 shows the flowchart of the selection process of the articles to be included in the present review.

## Results

**Fig. 2:**
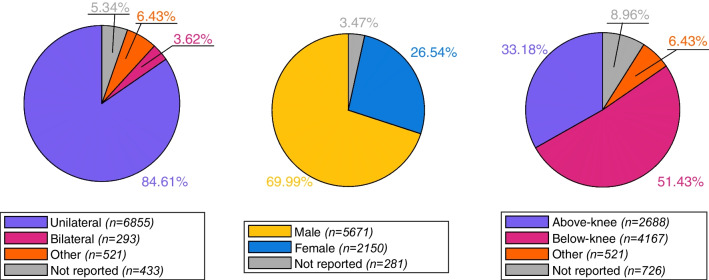
Demographics of the participant sample included in this review. Left: Distribution of unilateral, bilateral, or other amputations. Middle: Distribution of male and female participants. Right: Distribution of above-knee, below-knee, and other amputations

The total number of people with lower-limb amputation considered by the included articles was N = 8102, of which 84.61% (n = 6855) were unilateral, 3.62% (n = 293) bilateral lower-limb amputations, and 6.43% (n = 521) other amputations (above/below elbow), while in 5.34% (n = 433) the status was not reported. The majority of the participants were male (69.99%, n = 5671), while female participants represented 26.54% of the sample (n = 2150), and in 3.47% of the cases the sex was not indicated (n = 281). Regarding the amputation level, 51.43% (n = 4167) were below-knee (including foot amputation, and transtibial amputation), and 33.18% (n = 2688) were above-knee amputations (knee exarticulation, transfemoral amputation, hip exarticulation or hemipelvectomy). The remaining participants were affected by either an upper-limb amputation (6.43%, n = 521) or the amputation level was not reported (8.96%, n = 726). The demographics can be seen in Fig. 2.

**Fig. 3:**
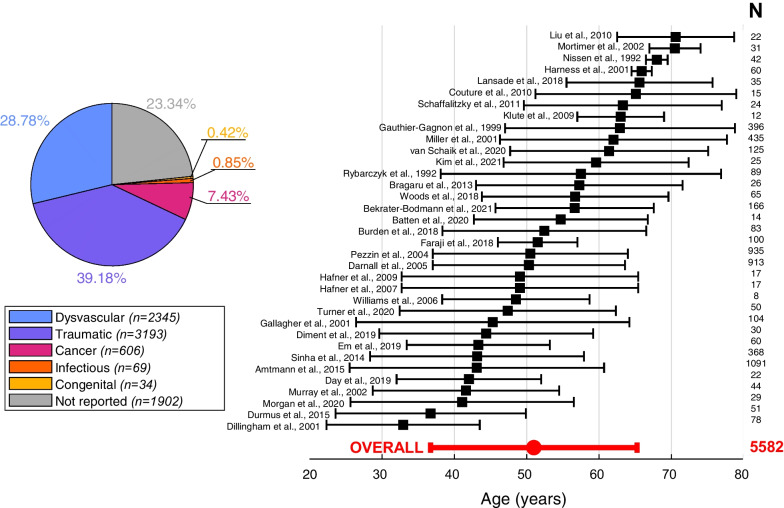
Left: Distribution of the causes of amputation. Right: Age distribution (mean ± standard deviation) in 35 papers from which it was possible to retrieve the age, and the weighted average for the whole sample, weighted by the number of participants in each study

The total number of amputations from the etiological perspective was N = 8149, because one paper allowed for multiple causes of amputation [71]. Of this sample, 39.18% were due to traumatic events (such as accidents or injuries, n = 3193), 28.78% were due to dysvascular reasons (such as peripheral vascular disease or diabetes, n = 2345), 7.43% were due to cancer (n = 606), 0.85% were due to infection (n = 69), and 0.42% due to congenital causes (n = 34). The remaining 23.34% of the causes were not reported or they were reported as “others” by the authors (n = 1902). The distribution of causes of amputation can be seen in Fig. 3. The prosthesis type (mechanical, microprocessor-controlled knee and ankle prostheses) was reported in only 1.49% of the cases (N = 121) [17, 52, 64, 74, 107].

The age of the overall subject sample was 50.98 ± 14.28 years, as shown in Fig. 3. The average age was calculated as a weighted average according to the sample size, from all papers included in the review which reported the age of their participants. The geographical origin of the participants was as follows: 61.21% from North America, 30.25% from Europe, and 7.42% from Asia, while the remaining participants were located in Africa or Oceania (remaining 1.12%).

**Fig. 4:**
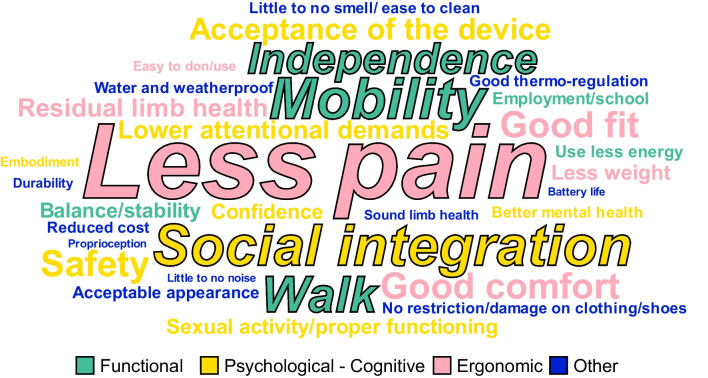
Word cloud of identified user needs. The larger the words, the more often the specific need was mentioned in the literature. The words with an outline represent the most commonly expressed user needs

Altogether, 31 different user needs have been identified in the literature and they are shown in the word cloud in Fig. 4, where the frequency of the occurrences of a specific need in the literature is proportional to the size of the word. In addition, the first 5 most frequently mentioned needs were highlighted (“Less pain”, “Mobility”, “Social integration”, “Independence” and “Walk”). However, it has to be noted, that this representation of the importance of user needs might be biased. The user needs that are popular and/or common in the research community might receive more attention in the literature, leading to a more prominent representation in the word cloud. Therefore, some needs that are important in real-life might be underrepresented in Fig. 4 simply because they are not yet in the focus of the research community. All needs mentioned in the literature were reported in this manuscript, except for those related to prosthetic service providers (e.g., the needs related to the users’ education about the alignment of prosthetic devices, or satisfaction with clinical practice [92], etc.). Such needs have been left out as they cannot be directly addressed by the development of prosthetic devices. The collected needs have been categorized into “Functional needs”, “Psychological and cognitive needs”, ”Ergonomic needs” and “Other needs” as shown in Table 1, which also indicates the relevant reference(s) mentioning the specific need as well as the estimated participant sample size. Functional needs reflect the physical activities which the users would like to do, and the requirements needed to perform those tasks. Psychological needs were identified as the mental and cognitive requirements expressed by the users related to the use of the device. Ergonomic needs are the requirements described by the users directly related to the interaction between their residual limb and the prosthetic device and the minimization of the risk of injury or harm. Other needs were defined as the requirements reported by the users that, however, did not fit into any of the previous categories and were mostly design-related.

**Table 1:** Collection of user needs expressed in the literature

	User needs	References	Sample size
Functional Needs	Walk	[34, 79, 84, 85, 87, 93, 96, 105–107]	697
	Use less energy	[79, 87, 106, 107]	520
	Independence	[34, 79, 80, 84, 85, 87, 96, 99, 108]	491
	Mobility	[17, 80, 82, 91–93, 95, 96, 99, 105, 107]	510
	Employment/school	[54, 80, 95, 99]	378
	Balance/stability	[17, 79, 91, 96, 107]	186
Psychological and cognitive needs	Safety	[17, 74, 81, 91, 96, 106, 107, 109]	959
	Confidence	[91, 93, 96, 107, 108]	118
	Integration in social/leisure activities/life	[34, 44, 54, 68, 79, 80, 84, 85, 95, 99, 105]	698
	Better mental health	[21, 64, 96]	988
	Sexual activity/proper functioning	[70, 83, 100, 103, 104]	286
	Acceptance of the device	[54, 79, 82, 85–87, 105]	374
	Lower attentional demands	[52, 81, 91, 93, 106, 107]	903
	Embodiment	[71, 108]	196
	Proprioception	[92]	12
Ergonomic needs	Good Comfort	[69, 72, 73, 79, 97, 98, 102, 108]	2889
	Good Fit	[72, 73, 91–93, 98, 102, 108]	805
	Residual limb health	[72, 79, 82, 85, 92, 105]	685
	Less pain	[54, 60, 63, 66, 67, 72, 73, 82, 84, 85, 87, 93, 94, 98, 101, 105]	2322
	Less weight	[54, 78, 79, 102, 107]	647
	Easy to don/use	[79, 92]	104
	Good thermo-regulation/less sweating	[88, 91, 92]	67
Other needs	Little to no noise	[79]	92
	Little to no smell/ease to clean	[65, 79, 92]	257
	Acceptable appearance	[1, 54, 65, 105]	334
	Sound limb health	[93, 106]	425
	Durability	[79, 92]	104
	Water and weatherproof	[85, 93, 107]	65
	No restriction/damage on clothing/shoes	[65, 79, 87]	267
	Reduced cost	[54, 82, 93]	120
	Battery life	[107]	10

### Functional needs

One of the most frequently expressed functional user needs in the literature is the ability to walk [34, 79, 84, 85, 87, 93, 96, 105–107]. This includes the ability to walk on streets and sidewalks, in close spaces or on slippery surfaces, and being able to climb stairs [79, 87]. Furthermore, lower limb prosthesis users with a lower mobility level (e.g. K2) desire to be able to walk on uneven terrain as well as on steep ramps, and generally to walk faster or take longer steps [107]. Also, during the use of a lower limb prosthesis, users express that they do not want to feel off-balance [79, 96], even during dynamic scenarios which require dynamic balance control [17] and stability [91, 107]. The socket design [110], prosthesis alignment [14, 111, 112], microprocessor-controlled systems, and artificial sensory feedback have all shown the potential to improve walking and balance behavior [113–116]. However, impairments in walking and balance are still widespread and remain a challenge in the field of lower-limb prosthetics [117, 118].

Another need that is expressed is the ability to walk independently [34]. Independence in general is a prominently expressed need [34, 79, 80, 84, 85, 87, 96, 99, 108]. This includes the ability to live an independent lifestyle, which in turn might be able to prevent low self-esteem or frustration [34, 96]. Being able to perform activities of daily living, including the ability to do transfers such as standing up and sitting down [17, 80, 99, 105, 107] or using transportation to go to the gym [85], helping with housework [80, 99], the ability to take care of someone else, and not being a burden to their partner or family [79, 87], are stated as important indicators of an independent lifestyle of a person with a lower limb amputation.

The functionality of a device assigned to the user strongly depends on their expected mobility level. Considering people with amputations due to vascular reasons, only less than a third of them would be able to ambulate outdoors. If the patient had limited functional abilities before surgery, the chances to walk afterwards are even lower [119]. Prosthesis users with higher mobility levels mentioned that they would like to be able to get in/out of the car [79, 99], have a knee joint that locks and unlocks reliably in more than one or two positions creating thereby more opportunities to use the device, be able to kneel, stoop, or to be free moving (e.g., when riding a bicycle [79, 93, 99]). Many would welcome a flexible ankle joint with adjustable alignment in the anterior–posterior or medial–lateral direction [79, 92]. A more versatile system that can support movements other than walking on the level ground might also facilitate independence [120–122]. For instance, a powered knee-ankle prosthesis can be used to actively support sit-to-stand and stand-to-sit maneuvers, thereby reducing the commonly observed asymmetry in the peak of the vertical ground reaction force between the sound leg and the prosthesis [123]. These advanced systems have allowed users to walk more naturally and descend slopes and stairs faster [74, 124, 125]. Such devices can improve the weight distribution and thereby contribute to maintaining the health of the sound limb, which is also a need expressed by the users [93, 106].

Being able to walk longer distances [106] goes hand in hand with the need for less energy required to ambulate with a prosthetic device, which is another prevailing need expressed in the literature [79, 87, 106, 107]. Lower limb prosthetic users report that they would like to fatigue less during the use of their prosthetic limb [106, 107], which is in line with findings in the literature regarding the inefficiency of the gait of people with lower limb loss [14, 126, 127]. Furthermore, users report that they often perform additional planning to reduce the amount of unnecessary ambulation with the prosthetic device and that it can be difficult to do spontaneous tasks, like responding to the phone or the doorbell [87]. The excessive physical effort when using the device or discomfort experienced while wearing the prosthesis impedes successful adaptation to the device [128]. Importantly, adding microprocessor-controlled knees or powered ankles has shown the potential to decrease the energy expenditure of the users [129–132].

Good mobility is a prerequisite for a lot of professions, and people with lower-limb amputations express the need to continue with their employment (or school in younger aged prosthetic users) post-amputation [54, 80, 95, 99]. The “return-to-work” rate represents the proportion of prosthetic users that continue working after amputation, and the studies report very different estimates, i.e., 48% [133], 60% [122], 66% [134], and 67% [135] of the people with lower-limb amputation returned to work within about a year, but often to physically less demanding tasks. The discrepancies in reported return-to-work rates are due to different definitions of successful employment, small sample sizes and subject characteristics (transtibial vs. transfemoral amputation, or different causes of amputation), different lengths of follow-up, and inconsistent information about the type of work before and after amputation [134]. Physical function and pain are significant predictors of return to work [136], but the factors regarding the work environment are also important. The highest chance of returning to work is when the work is less physically demanding, and when the time worked at the place before the injury was long [136]. Similarly, a reduction in leisure activities with higher physical demands is seen after amputation, including crafts, outdoor activities, and sports [44]. The productivity loss of the employees due to missed work hours is reduced in the users of microprocessor-controlled devices [137].

### Psychological and cognitive needs

The perception of safety while using a prosthesis is reported as the most important need, which is independent of the mobility level of the people with lower limb amputation [17, 81, 91, 96, 106, 107, 109]. Lower limb prosthetic users wish for a better, more sensitive stumble recovery [17] and a reduced fear of falling [81, 106], including a reduced occurrence of falls [74, 107]. The consequences of a fall are not purely physical (injuries), but can also be psychological (embarrassment, or avoidance of risky activities in the future [91, 96]). In addition, lower limb prosthesis users express the need for more confidence in their prosthetic limb, which is reflected in the willingness to increase the proportion of time spent on the residual limb during single stance and/or greater confidence in managing new terrain or places [107]. The need for more confidence is also related to an increased perception of the value of prosthesis use [108]. The use of microprocessor-controlled knee joints can improve balance and balance confidence [107, 115] while reducing falls and stumbles [124, 138]. Some studies report no significant reduction in falls, but the users still expressed increased balance confidence [139, 140].

Another critical psychological need is the integration in social life [34, 44, 54, 68, 79, 80, 84, 85, 95, 99, 105]. Lower limb prosthesis users report being worried about feeling helpless, isolated, or excluded from others [34, 68, 85], or feeling social discomfort and a gap between them and their surroundings [54], especially if they are unable to participate in an active social life outside of their home [79, 80, 99]. And indeed, the users often emphasize affective (emotional) and social constraints to participate in certain leisure activities [44]. Being able to return to social activities is associated with self-fulfillment [54]. In the worst cases, the unmet need for an active social life can result in depression [80], or problems in the user’s marital life or with friends. The perception of the personal body image is an important factor when it comes to social reintegration [141]. The appearance of the prosthesis has been positively associated with overall prosthesis satisfaction [105]. The positive body image can motivate prosthesis users to join/re-join the workforce or engage in social activities, courtship, dancing, or even wearing gender-specific attire, such as high heels [64, 142]. The opposite effects might occur when the aesthetics of the prosthesis are unpleasant to the user and the ones around it, for instance, in the case of gender-inadequate limbs [142]. Furthermore, anxiety and sleep disturbances are reported [64]. The users state that it is important for themselves as well as for their partners or others in their surroundings to accept the prosthetic device [54, 79, 82, 85–87]. The acceptance of the device is related to the need for sexual activity or proper sexual functioning [70, 83, 100, 103, 104], predominantly reported by male users, because body image disturbances [1] and self-consciousness can lead to sexual dysfunction [103].

Lower attentional demand while using a prosthetic leg is another frequently reported need [52, 81, 91, 93, 106, 107]. The users state that they need to concentrate on each step while walking [81, 106], whereas they desire the capacity to walk and think about other things or walk and talk on the phone at the same time [107]. Being able to walk without thinking is related to higher balance confidence [143], which reinforces the intertwined relationship between the different user needs [51]. The fact that people with lower-limb loss have to concentrate on each step has been associated with increased fear of falling [39], and vice versa, the improvement in balance confidence has been shown to lead to better multitasking ability [144]. While dual-tasking during walking and standing showed worse performance for people with lower-limb amputation compared to able-bodied subjects, this effect could be reduced by the use of microprocessor-controlled knees [145]. Advanced prosthetic devices may therefore reduce the cognitive demand and increase the ability to multitask [74]. Moreover, there seems to be a relationship between the general cognitive profile (e.g., existing cognitive impairments) and the ability to perform certain tasks or participate in activities of daily living [51]. Recent research has shown that there is a relationship between cognitive abilities and the perception of the body image of a person with amputation [146].

Lastly, the users report the need for feeling less disconnected from their leg, which is reflected by the fact that they refer to the prosthesis as “the leg” instead of “my leg” [108] and the expressed need for proprioceptive feedback, i.e. perceiving the motion of their bionic foot and interaction with the environment [92]. Therefore, there is a need for a better embodiment of prosthetic systems, which is closely related to the appearance and the functioning of the device [71]. The lack of embodiment can be an important factor in the decision to reject a prosthesis [71]. Yet, many of the reasons cited as motives for the rejection of prosthetic devices are related to the initial experiences/impressions during the adaptation period, whereas the users who persevere in the discomfort often report a much more natural and embodied experience in the later phase [142]. Furthermore, the frequency and amount of prosthesis use are shown to be positively correlated with the accuracy of estimating the prosthetic limb length, which is, in turn, an indicator of embodiment [147]. Reducing sensory conflicts and providing artificial sensory feedback could enhance embodiment [148–150] and this can be therefore a promising solution to improve device acceptance. Similarly, the cosmetic resemblance between the prosthesis and the biological limb has been used to evaluate the integration of the device in the users’ body scheme [150].

### Ergonomic needs

Good comfort and fit of the prosthetic leg have been identified as one of the most prominent user needs in several studies [69, 72, 73, 79, 97, 98, 102, 108], and this includes comfort during specific activities such as standing and sitting [79]. According to the literature, 57% of prosthesis users are dissatisfied with the comfort of their prostheses, and over 50% report pain during use [72, 73]. Furthermore, comfort and hence satisfaction often decline over time [151]. Socket fitting and design are considered one of the most important features of a prosthetic device [72, 73, 91–93, 102, 108] as the socket is the location where forces are being applied [98, 152]. Improving the fitting can increase self-reported perceptions of comfort [153]. The quality of fitting not only influences comfort [154] but also the performance of a person with an amputation during locomotion, such as energy consumption, walking velocity, and gait symmetry [155]. Ultimately, if the socket is uncomfortable, the user may not wear the prosthesis [154]. A good example of an improvement in comfort thanks to technology is the introduction of Total Surface Bearing sockets compared to Specific-Surface Bearing. The Total Surface Bearing technology allowed the spreading of the load over the entire residual limb rather than a localized area, which removed local stresses and enhanced comfort [156]. In addition, more than 53% of the prosthesis users report feeling discomfort due to excessive heat and sweating, which can be easily triggered by even a small increase in temperature (~ 1 to 2 °C) [89]. Different materials have been developed to improve the socket thermoregulation capacity [110].

The users expressed the need for a prosthetic device that is easy in donning and doffing and use in general [79, 92]. Improvements in donning are usually associated with fitting and mounting technology as well as with device comfort. Most recent suspension systems are using subatmospheric pressure mechanisms to ensure a comfortable and proper fitting: distal locking mechanisms (pin-lock, magnetic-lock, and lanyard strap), liner-fit suction (vacuum-assisted sockets, unidirectional valve), hypobaric and skin-fit suction [110]. Vacuum-assisted sockets have been experienced as difficult to don in some cases [157]. Magnetic-lock suspension systems have shown to be easier to don-doff compared to traditional pin-lock and suction systems [158], even though the users of pin-lock have reported an overall high satisfaction [157, 159–162]. Seal-in liners are associated with problems with donning and doffing but the users also express high satisfaction overall [159, 163]. Indirect causes can also lead to problems related to donning, for instance, volume increase from 3 to 5% in the residual limb has shown to increase the difficulties to don the device [164].

The health of the residual limb is another important user need [72, 79, 82, 85, 92, 105], and it includes avoidance of skin irritation (e.g., blisters, sores or rashes, and ingrown hairs) [72, 79]. Furthermore, the users state that they would like a pleasant feeling and smooth texture of the prosthesis against the residual limb [79], and the use of the device without any pain [54, 60, 63, 66, 67, 72, 73, 82, 84, 85, 87, 93, 94, 98, 101, 105]. This includes residual limb pain, phantom limb pain, or other pain such as in the lower back area. The perception of pain is a widely reported side-effect of prosthetic use [72, 73] but might be reduced by an optimized fitting of the device. Improving the interface might not only decrease the stump pain but it can also improve other socket-related issues such as biomechanical functionality and control [110]. Phantom limb pain could be potentially addressed by the addition of artificial sensory feedback, as it has been shown that the feedback can alleviate pain in lower-limb prosthesis users when using both invasive [148] and non-invasive stimulation strategies [165, 166].

The users desire to reduce the weight of the prosthetic device [54, 78, 79, 102, 107]. The heaviness of the prosthetic leg constitutes one of the main causes of rejection [55]. Surprisingly, the users perceive their leg as being too heavy [54], even if the prosthetic leg weighs less than its biological counterpart [167]. The perception of the weight of the prosthetic device is influenced by multiple factors [168], such as previous experience and expectations, or muscle contraction and feedback from peripheral receptors. Indeed, providing sensory feedback has been shown to reduce the perceived weight, which is also an indicator of prosthesis embodiment [149].

Another ergonomic need expressed by the users was better thermo-regulation [88, 91, 92] and thermal-related discomfort was reported by at least half of the users [89]. However, there is also evidence that the skin temperature of the residual limb does not explain the experienced thermal discomfort and is therefore not an appropriate predictor of thermoregulatory issues [88].

### Other needs

Generating little to no noise, smell [79] and easy cleaning [65, 92] are also important requirements stated by prosthesis users. The noise in a prosthesis is often interpreted as an indication of a mechanical problem that needs to be fixed [79]. The technological improvements have been successful (to a certain extent) in decreasing the noise level without sacrificing performance [169]. Odors in the socket can arise due to bacterial invasion: to address this, textile spacers with bacteriostatic fibers have been added to reduce the odors but this also allows better breathing of the stump [170]. Similarly, silver antibacterial particles have been added to reduce bacterial concentrations in the socket [171]. The ease of cleaning the device is associated with other needs, such as being waterproof for example.

The users would like that their prosthesis is durable [92], especially regarding the cover [79]. A broken device increases the chance of the user becoming dependent on others [172]. Liners’ durability has improved over time [110, 173]. While the liner can be easily replaceable and is less likely to lead to the occurrence of a dramatic event, the breaking or malfunction of the prosthesis increases this probability. In a study from 1996, the authors indicated that 12% of the falls were due to prosthesis-related issues [174].

Another important aspect is the ability of the device to assist the user in every situation [107], for instance, weatherproof devices are desired by the users [85, 93]. These devices allow the users to perform outdoor activities and in some cases even swim or shower with the device. A design-related need is that there are no restrictions imposed on the choice of clothing or shoes [65, 87] and that the clothing is not damaged by the interaction of the prosthesis cover during use [65, 79].

Other expressed needs are sufficient battery life [107], and a lower cost of the device [54, 82, 93]. Users complain that they have to charge their microprocessor-controlled prosthetic devices every night [107]. Sufficient battery life can be obtained by optimizing the energy efficiency of the actuators. Low-impedance actuators, for example, can store energy during phases of negative joint work and recharge the leg’s batteries [169]. There is a tradeoff between cost and function [175]. While overall expenses related to amputation are reduced thanks to microprocessor-controlled systems, the overall costs remain high [137]. In cases where insurance does not cover the costs, this includes expenses for the device itself, but also related costs for maintenance of the device, changing sleeves, or residual limb skin care products [54].

## Discussion

### User needs are interrelated and multifaceted

This review has identified a comprehensive collection of user needs that are not yet fully addressed with current commercial lower limb prosthetic devices. Therefore, it is important to translate these needs into critical guidelines for the development of user-centered lower-limb prosthetic systems. However, some challenges should be addressed for this translation (from user needs to design specifications) to be successful. The identified needs are clearly multifaceted; they are context-dependent and there is a complex interaction between different groups of needs as well as between specific needs and overall experience with the system and improvement in the quality of life.

**Fig. 5:**
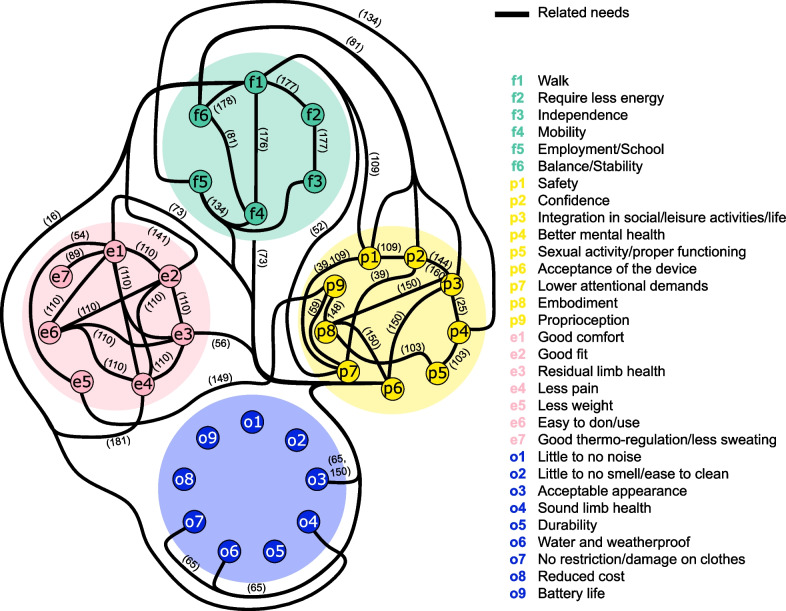
Map of intrinsically related needs of lower-limb prosthesis users. Individual functional (top, green), psycho-cognitive (right, yellow), ergonomic (left, pink), and other needs (bottom, blue) are connected using solid black lines to denote the intrinsic relationship between the user needs, as expressed in the literature. The number on each line refers to the reference that established the relationship. For example, the needs e1 (good comfort) and e7 (good thermo-regulation/less sweating) have been connected through the reference [89] as the authors indicated that more than 53% of the prosthetic users are expressing discomfort due to excessive socket heat or sweating

Needs are often intrinsically related, i.e., the needs stated by the users are not isolated, as the literature often reports the dependency between the needs. Such relationships between the identified user needs have been summarized in Fig. 5, and are seen across ‘functional’ [81, 134, 176–178], ‘ergonomic’ [54, 89, 110], ‘psycho-cognitive’ [25, 39, 59, 103, 109, 144, 148–150] and ‘other’ groups [175, 179, 180]. The category of “Other” needs is less interrelated, which indicates that these relations still remain to be investigated and established. However a trade-off between features such as weight, noise, smell, appearance, durability, ability to be waterproof/weatherproof, restriction/damage on clothes, battery life and costs have to be considered [175, 179, 180]. Indeed, the selection of materials and prosthesis features impacts the overall prosthesis cost differently. For example, the durability, weight, and cost of the prosthesis material are critical aspects to manage when the prosthesis is addressed in lower- to middle-class economies [180]. The needs are not only related within the same group but also across groups. For example, socket fitting is a prominent aspect from a comfort perspective [110], but it also critically impacts functioning [181]. Similarly, the embodiment of the prosthetic device is related to acceptance of the device, but it can also strongly facilitate participation in everyday life and involvement in social activities [150]. Therefore, the close interaction of different needs might indicate that multiple paths can be pursued to substantially impact the users’ quality of life. In addition, as explained below, the strength of the relations between the needs might differ across individuals as well as time instants during the use of the device.

While functional improvements are important, Fig. 4 clearly shows that the needs most frequently reported by the users, and therefore being identified as very important, belong to the psychological and ergonomic domain (i.e., less pain and social integration). Although related, such needs cannot be completely “reduced” to the functional capabilities of the device. The improvements in the socket or liner technology, or overall appearance have been shown to improve comfort, fit, residual limb health, smell and noise, the ease to don or use the device, and durability. Because these needs are influenced by a large number of factors, other than the design process itself, it is possible that they can never be completely fulfilled by the development of new devices, if they focus on improving function only. Such devices are at risk to be rejected, although potentially useful, because they do not address the more prominent needs in the psychological and ergonomic domains.

The functional requirements and expectations about the prosthetic device highly depend on the characteristics of a specific user, such as his/her mobility level, age, health, and gender. For example, younger people (< 65 years old) assigned higher significance to the ability to walk in close spaces and the ability to walk on slippery surfaces [79] compared to the older generation. Some activities are more impacted as age increases [182], and it is reported that functional needs change with age. Specifically, independence when performing activities of daily living, social functioning, and general activities decrease with age. On top of that, functional abilities are negatively associated with other age-related factors such as dementia and renal disease. These factors often limit the functional abilities and fitness before the amputation and make post-surgical ambulation ten times less likely [183]. Female prosthetic users rated several functional needs with higher importance compared to males. These include: not feeling off-balance while using the prosthesis, the ability to walk with the prosthesis, ease of donning, and energy required to use the prosthesis [79, 119]. Thus, needs might be associated with specific user characteristics (such as age or gender) and differ between individuals [184].

These insights suggest that the development of new devices should consider the ability to customize the device to a specific subject or user group (e.g., elderly or younger, male and female). Ideally, the system should also “recognize" the evolving needs of a specific user, and enable a “spiral of adaptation”. Such a device would be able to adapt to the needs as they change during the user’s lifetime depending on the previously identified factors, while the user simultaneously adjusts to the conditions of the new interface [120]. Correct identification of the current user needs at a specific time during the device use is necessary to adapt the device optimally; for example, improving only the controller of a prosthesis does not necessarily translate into an increased quality of life [121]. This indicates that either more factors related to assessment and/or development have to be considered, or that the specific improvement might not affect all the participants equally. As a result, setting the right focus in developing prosthetic devices concerning user needs can be seen as critical and it requires balancing various areas in the lives of those affected by lower limb loss (functional, psychological, cognitive, ergonomic, and others). A system that would strike such a balance by successfully addressing these different aspects might lead to the best possible solution for future prosthetic devices.

### Further technological developments to address needs across domains

As described in the results section, the development of mechatronic lower limb prostheses has had an impact across several categories of the user needs domain. For example, these systems provided the ability to walk with less energy [129–132], increased mobility [121] and independence [120–122], higher productivity at the workplace due to a lower number of missed days [137], improved balance and stability [107, 115, 140], increased safety and confidence [107, 115, 140], decreased attentional demands [52, 74], enabled better sound limb health [106], and reduced cost (neglecting purchasing price of the devices) [137]. However, there is a room for further critical improvements, especially in the following areas:

- The ability to walk and balance is still impaired with respect to able-bodied population [117, 118]
- Only a few fully powered devices exist to support the people with lower limb loss, especially during activities like standing up from sitting down, which reduces overall independence and mobility [123]
- While balance confidence has improved, the actual number of stumbles and falls did not significantly decrease [139, 140]

In addition to improvements in the control of prosthetic devices, artificial sensory feedback has been shown to address some of the user needs. These include increased gait symmetry [185, 186], embodiment [148], reduced phantom limb pain [59, 166], and reduced weight perception [149]. With the further development and tuning of feedback parameters, these aspects could be potentially improved even more. However, there is still a lack of commercially available solutions for artificial sensory feedback.

What remains unknown, is how to effectively address aspects related to integration into social life, participation in sexual activity, overall acceptance of the device, appearance, battery life, and restrictions imposed on clothing and shoes. These aspects are especially difficult to generalize across the overall population of people with lower limb amputation, which makes the integration into the development process and finding a successful solution challenging.

### Limitations of current assessment methods

The contributions of new developments in the field of mechatronic prosthetic devices could have the potential to generate real benefits for end-users. Nevertheless, to establish the latter objectively, it is paramount to evaluate qualitatively and quantitatively if technological improvements are translated into increased well-being for users. However, the current evaluation methods are likely to be insufficient for that goal, which thereby motivates the formulation of novel assessment approaches.

Ultimately, the individual user needs to be met by prosthetics development should be seen through the prism of a better overall quality of life [187]. Several predictors have been associated with a decreased quality of life of people with amputation: condition of the residual [188, 189] and sound limb [188], fit of the prosthesis [189], time since amputation [141], walking distance [188], mobility problems [190], social support [141] and depression symptoms [22]. The indicators of mental health such as depression and anxiety in lower-limb prosthetic users have been related to a multitude of other factors: pain occurrence [21], length of inpatient stay [25], cause of amputation [20], presence of comorbidities [21, 25, 26, 28], social support [68] and social discomfort [27, 32], and body image disturbances [31], etc.

Current assessment methods can be classified as self-report, professional report, or performance-based measures. Self-reported outcomes often do not correlate with objective measures of function, which highlights the need to include both or use a more holistic approach [191, 192]. What people perceive to be capable of doing, and what they actually achieved, seem to be somewhat disconnected [191]. Self-reports are also intrinsically limited regarding the driving of development, as the users need to imagine functions that do not yet exist. Therefore, when asked, many users state that they would not change anything about their prosthesis or they cannot think about something that would improve the functionality of the prosthetic device [79].

This highlights the need for an improvement in questionnaires to assess the needs of people with lower-limb loss. Ideally, such questionnaires should elicit the user needs in the form that can be translated into the requirements for the development of lower limb prosthetic devices. The surveys should give the users freedom to express their needs and they should also be generalizable across the population of prosthetic users. The minority of the included references reported the prosthesis type, which makes the generalizability of the expressed needs even more difficult. Some of the included references have used semi-structured interviews (e.g., [34, 44, 84, 85, 91, 93, 96, 97, 100]), which allow gaining a deeper understanding of aspects covered during the interview process. It is possible, however, that they lack objectivity, as the answers can be biased by the way the questions are being asked and/or by the attitude of the interviewer towards the participants. Another disadvantage is the fact that results are sometimes based on a small or biased (e.g., mostly males) sample, and might not be generalizable [84]. Another approach seen in the literature was the use of workshops with focus groups and subsequent sessions based on the Water Cooler Logic process. This process facilitates, guides, and documents open-ended discussions on the needs that were identified during the workshop focus group session. This approach was able to directly produce suggestions for future prosthetic device development, based on the needs expressed by the users [92].

While the prescription of prosthetic devices is mainly supported by objectively measurable outcomes, the actual use of the prosthesis is dependent on individuals’ impressions about their functional abilities. As a result, future research should account for both of these aspects (i.e., objective measurements and the individual’s impression), as well as for inter-subject differences between prosthesis users. An additional challenge is that performance-based assessment (objective evaluation) does not always correlate with subjective impressions. For instance, the use of a microprocessor-controlled knee can improve the subjective experience regarding cognitive burden compared to a conventional passive system, i.e., the users reported that they used less attention while using the former device [52]. However, the decrease in cognitive burden could not be confirmed with objective measurements (dual-tasking performance) [52].

The use of alternative assessment methods could help bridge this gap. The exploration of neural correlates (especially cortical activity), for example, showed that individuals using non-microprocessor-controlled prosthetic legs exhibited higher brain activity compared to subjects using microprocessor-controlled devices and the able-bodied population [193, 194]. Visual sampling can be as well related to a higher cognitive load, as the time spent looking at something is generally associated with the amount of cognitive processing. As shown in [195], visual sampling during a challenging task, such as stair climbing, is increased in people with amputation compared to healthy controls. This measure might be also related to other needs (e.g., confidence and safety), as it has been shown in able-bodied participants that there is a correlation between the perception of safety during stair descent and the need for the visual sampling of the stairs [196].

Different measurement tools have been used to quantify functional and mobility limitations or quality of life of prosthetic users [197, 198]. However, the tools often focus on only one aspect of the functional, psychological, or cognitive domain and fail to assess the impact of a prosthetic device holistically. Furthermore, it is nearly impossible to foresee long-term effects on functional abilities and prosthetic use with the current assessment tools [199–201].

Mapping the use of the prosthesis in daily life is critical to assess its impact holistically, in terms of overall user experience and quality of life. However, there is a limited knowledge of the functional benefits of prostheses use outside of a laboratory. Objective assessment tools should be more ecologically valid and not only lab-based but more related to real activities in the daily lives of those affected by the lower-limb loss. According to the international classification of functioning, disability, and health, evaluating prosthesis performance in the actual user environment (home, work) is an important component of functionality [202]. Clinical gait analyses are typically performed in a laboratory using optoelectronic motion capture systems and force plates [203]. These measurement techniques are considered the golden standard in movement analysis; however, they are not representative of the performance of people with lower limb loss in the real world, because of the limited space and constraints in terms of tasks that can be performed (pathways and surfaces) [203]. Another limitation is that single-session lab tests neglect the training effect [204].

## Conclusion

Amputation is a traumatic experience that has a substantial impact on the quality of life of those affected. Consequently, the users of prosthetic limbs express a diverse set of needs across functional, psychological, cognitive and ergonomic domains. They desire to maintain an independent lifestyle and actively engage in the tasks of daily life. They would like to come back to work and participate in social and leisure activities. To achieve such goals, the users expect to be able to safely stand (balance) and walk comfortably and efficiently with their prostheses across different surfaces and conditions. They would also prefer to invest less cognitive effort while using the prosthesis so that they can attend to different activities in parallel, like able-bodied individuals (“walk and talk”). However, user needs do have high variability, they interact and depend on other factors such as mobility level, health condition, age, and sex. They are also challenging to assess objectively with the current evaluation methods that are confined to the lab environment. The objective assessment often does not match the user self-report nor does it correlate with the overall satisfaction with the device and the quality of life. The aforementioned challenges will need to be addressed to understand and capture the user needs, especially as they evolve during the use of the device. These are critical parameters for driving the development of novel prosthetic solutions.

## Data Availability

Data sharing does not apply to this article as no datasets were generated or analyzed during the current study.
